# Supplementary material for: Estimates of the cost to build a stand-alone environmental surveillance system for typhoid in low- and middle-income countries
Source: PLOS Glob Public Health. 2023 Jan 26;3(1):e0001074. doi: 10.1371/journal.pgph.0001074 (PMC10021573; doi:10.1371/journal.pgph.0001074)
Supplement: S1 Text — Table A in S1 Text. Methods-based laboratory equipment requirements. Table B in S1 Text. Capacity of laboratory equipment. Table C in S1 Text. Unit costs for consumables. Table D in S1 Text. Operational paratmers for estimating labor-hours. Table E in S1 Text. Additional rates and values used in the cost model. (DOCX) [file pgph.0001074.s001.docx]

# **S1 Text: Cost Model Input Parameters**

**Table A.** Method-based laboratory equipment requirements. The number indicates how many of each piece of equipment is needed for a given aspect of the environmental surveillance method protocol. Equipment needs by method were obtained through surveys of participating laboratories.

| Equipment | Filter cartridge | Grab enrichment | Moore swab | Dead-end ultra-filtration | Differential Centrifugation | Membrane filtration | Tangential flow ultra-filtration | Enrichment | DNA extraction | Culture | qPCR | qPCR TAC | |
| --- | --- | --- | --- | --- | --- | --- | --- | --- | --- | --- | --- | --- | --- |
| IDEXX Sealer | 1 | 1 | 1 | 1 | 1 | 1 | 1 | - | - | - | - | - | |
| pH meter | 1 | 1 | 1 | 1 | 1 | 1 | 1 | - | - | - | - | - | |
| Thermometer | 1 | 1 | 1 | 1 | 1 | 1 | 1 | - | - | - | - | - | |
| PCR machine | - | - | - | - | - | - | - | - | - | - | 1 | 1 | |
| Centrifuge standard | 1 | - | - | - | 1 | - | - | - | - | - | - | - | |
| Centrifuge TAC | - | - | - | - | - | - | - | - | - | - | - | 1 | |
| Shaker table | 1 | - | - | - | - | - | - | - | - | - | - | - | |
| Pump | - | - | - | 1 | - | - | 1 | - | - | - | - | - | |
| Mini-spinner | - | - | - | - | - | - | - | - | - | - | 2 | 2 | |
| Water bath | - | - | - | - | - | - | - | - | 1 | - | - | - | |
| Chemical fume hood | - | - | 1 | 1 | 1 | 1 | - | - | - | - | - | - | |
| Incubator | - | 1 | 1 | 1 | 1 | 1 | - | 1 | - | 1 | - | - | |
| UV PCR hood | - | - | - | - | - | - | - | - | - | - | 1 | 1 | |
| Vacuum filtration unit | - | 1 | 1 | - | - | 1 | - | - | 1 | - | - | - | |
| Pipet-Aid | 1 | 1 | 1 | 1 | 1 | 1 | 1 | 1 | - | - | - | - | |
| Pipettor | 2 | - | 1 | 1 | 1 | 1 | 1 | 1 | 3 | 2 | 4 | 4 | |
| -80C freezer | 1 per lab, regardless of methods | | | | | | | | | | | | |
| -20C freezer | 1 per lab, regardless of methods | | | | | | | | | | | | |
| Balance | 1 per lab, regardless of methods | | | | | | | | | | | | |
| Hot stir plate | 1 per lab, regardless of methods | | | | | | | | | | | | |
| Glassware | 1 per lab, regardless of methods | | | | | | | | | | | | |
| Refrigerator | 1 per lab, regardless of methods | | | | | | | | | | | |  |
| Computer | 1 per lab, regardless of methods | | | | | | | | | | | |  |
| Biosafety UV cabinet | 1 per lab, regardless of methods | | | | | | | | | | | |  |
| Vortexer | 2 per lab, regardless of methods | | | | | | | | | | | |  |
| Autoclave | 1 per lab, regardless of methods | | | | | | | | | | | |  |
| Micro-centrifuge | 1 per lab, regardless of methods | | | | | | | | | | | |  |

**Table B.** Unit costs for consumables. Disposable costs include all supplies and wastage related to the given method, as reported via surveys of participating laboratories. All values in US dollars, 2019. For those without an adequate sample size (n<3), we used the mean reported value as the median in the simulated distribution and assumed a 20% width for the 25^th^ and 75^th^ percentiles.

| Value | | Filter cartridge | Grab enrichment | Moore swab | Dead-end ultra-filtration | Differential Centrifugation | Membrane filtration | Tangential flow ultra-filtration | Enrichment | DNA extraction | Culture | qPCR | qPCR TAC |
| --- | --- | --- | --- | --- | --- | --- | --- | --- | --- | --- | --- | --- | --- |
| Disposables | 25^th^ | 60.00 | 1.50 | 0.80 | 283.50 | 0.80 | 27.00 | 51.50 | 2.50 | 1.80 | - | 2.00 | 45.00 |
|  | Median | 70.00 | 3.00 | 1.00 | 315.00 | 1.00 | 30.00 | 102.00 | 4.50 | 10.00 | - | 11.00 | 55.00 |
|  | 75^th^ | 80.00 | 4.50 | 1.20 | 346.50 | 1.20 | 33.00 | 103.00 | 17.00 | 20.00 | - | 20.00 | 65.00 |
| Reusables, annual per team | 25^th^ | 8.50 | | | | | | | | | | | |
|  | Median | 17.00 | | | | | | | | | | | |
|  | 75^th^ | 117.00 | | | | | | | | | | | |
| Reusables, daily per team | 25^th^ | 1.50 | 1.50 | - | - | 1.50 | 9.00 | 51.50 | - | - | - | - | - |
|  | Median | 3.00 | 3.00 | - | - | 3.00 | 18.00 | 103.00 | - | - | - | - | - |
|  | 75^th^ | 4.50 | 4.50 | - | - | 4.50 | 48.00 | 103.00 | - | - | - | - | - |
| Sample size |  | 2 | 1 | 2 | 1 | 1 | 2 | 1 | 4 | 7 | NA | 4 | 1 |

**Table C.** Rates and values used in the cost model across protocols, as they were not directly tied any specific method. Additional parameters that were used in the cost model that were used across the protocols and were not directly tied any specific methods.

| Parameter | Value | Reference |
| --- | --- | --- |
| Equipment maintenance rate | 4% | Assumed |
| Truck maintenance rate | 16% of depreciation | Calculated per Republic of South Africa Government Gazette [14] |
| Truck annual operations cost rate | 35% of depreciation | Calculated per Republic of South Africa Government Gazette [14] |
| Daily pay rate for technicians | $3.50, $7.00, $40.00 | Survey of participating labs (25^th^, median, 75^th^ percentiles) |

**Table D.** Capacity of laboratory equipment. Daily capacity represents the maximum number of samples that could be processed by that piece of equipment in a single day. Lifespan (samples) represents the maximum number of samples that could be processed by that piece of equipment before it needs to be replaced. Lifespan (years) represents the maximum number of years that a piece of equipment could be in place before it would need to be replaced; duration is consistent with the US IRS guidance on depreciation. Values were obtained through surveys of participating laboratories and online research for each piece of equipment.

| Equipment | Daily capacity (samples) | Daily capacity (teams) | Lifespan (samples) | Lifespan (years) |
| --- | --- | --- | --- | --- |
| IDEXX Sealer | - | - | 5,000 | - |
| pH meter | - | - | 1,000 | - |
| Thermometer | - | - | 5,000 | - |
| PCR machine | 300 | - | - | 5 |
| Centrifuge standard | 50 | - | - | 5 |
| Centrifuge TAC | 50 | - | - | 5 |
| Shaker table | 50 | - | - | 5 |
| Pump | 10 | - | - | 5 |
| Mini-spinner | 300 | - | - | 5 |
| Water bath | 500 | - | - | 5 |
| Chemical fume hood | 1,000 | - | - | 5 |
| Incubator | 100 | - | - | 5 |
| UV PCR hood | 600 | - | - | 5 |
| Vacuum filtration unit | 500 | - | - | 5 |
| Pipet-Aid | 600 | - | - | 5 |
| Pipettor | 600 | - | - | 5 |
| -80C freezer | 20,000 | - | - | 5 |
| -20C freezer | 15,000 | - | - | 5 |
| Balance | 1,000 | - | - | 5 |
| Hot stir plate | 1,000 | - | - | 5 |
| Glassware | 1,000 | - | - | 5 |
| Refrigerator | 300 | - | - | 5 |
| Computer | 10,000 | - | - | 5 |
| Biosafety UV cabinet | 600 | - | - | 5 |
| Vortexer | 600 | - | - | 5 |
| Autoclave | 200 | - | - | 5 |
| Micro-centrifuge | 100 | - | - | 5 |
| Truck | - | 1 | - | 7 |

**Table E.** Operational parameters used for estimating the number of labor-hours required to collect and process samples. Active lab staff time in hours. Blanks are spaces where the data are not applicable; these are not methods associated with collection. Values obtained via surveys of participating laboratories.

| Value | | Filter cartridge | Grab enrichment | Moore swab | Dead-end ultra-filtration | Differential Centrifugation | Membrane filtration | Tangential flow ultra-filtration | Enrichment | DNA extraction | Culture | qPCR | qPCR TAC |
| --- | --- | --- | --- | --- | --- | --- | --- | --- | --- | --- | --- | --- | --- |
| Samples collected per team per day | Min | 1 | 2 | 2 | 1 | 2 | 2 | 2 | - | - | - | - | - |
|  | Mode | 2 | 5 | 5 | 2 | 5 | 5 | 5 | - | - | - | - | - |
|  | Max | 4 | 10 | 10 | 4 | 10 | 10 | 10 | - | - | - | - | - |
| Technicians per collection team | | 3 | 2 | 2 | 2 | 2 | 2 | 2 | - | - | - | - | - |
| Laboratory batch size | | 6 | 12 | 20 | 6 | 16 | 6 | 6 | 20 | 12 | 20 | 80 | 8 |
| Active lab staff time, per batch (hours) | Min | 1.5 | 0.5 | 0.3 | 0.5 | 1.0 | 0.5 | 1.5 | 0.3 | 1.0 | 0.5 | 1.0 | 1.0 |
|  | Mode | 2.0 | 1.0 | 0.5 | 1.0 | 1.5 | 2.0 | 2.0 | 0.5 | 2.0 | 1.0 | 2.0 | 2.0 |
|  | Max | 3.0 | 2.0 | 1.0 | 2.0 | 2.0 | 6.0 | 3.0 | 1.0 | 4.0 | 2.0 | 3.0 | 3.0 |

**Additional References**

14. Republic of South Africa Government Gazette. [Cited October 12, 2020]. Available from: https://www.sars.gov.za/Tax-Rates/Employers/Pages/Rates-per-kilometer.aspx
