# Supplementary material for: Estimates of the cost to build a stand-alone environmental surveillance system for typhoid in low- and middle-income countries
Source: PLOS Glob Public Health. 2023 Jan 26;3(1):e0001074. doi: 10.1371/journal.pgph.0001074 (PMC10021573; doi:10.1371/journal.pgph.0001074)
Supplement: S2 Text — (DOCX) [file pgph.0001074.s002.docx]

# **S2 Text: Survey on Collection, Concentration, and Assay Methods for Environmental Surveillance of Salmonella Typhi**

**Sample type**

🞏 Drinking water 🞏 Wastewater 🞏 Surface water 🞏 Other

**Volume considerations**

Average volume collected: mL

*Collection method*

- Grab sample

🞏 Composite sample

🞏 Method (pump, manual, Moore swab)

🞏 Other

*Concentration*

- Concentration factor

(initial volume/final volume):

🞏 Discrete volume processed (*e.g.*, filtration)

Sample volume concentrated L

Final concentrate volume: mL

🞏 Unknown vol. processed (*e.g.*, Moore swab)

Final concentrate volume: mL

**Primary concentration**

🞏 Yes

🞏 BMFS 🞏 Membrane filtration 🞏 Centrifugation 🞏 Moore swab

🞏 Dead end ultrafiltration 🞏 Tangential flow ultrafiltration

🞏 Other

🞏 No

**Secondary concentration**

🞏 Yes

🞏 PEG precipitation

🞏 Skimmed milk flocculation

🞏 Other

🞏 No

**Enrichment**

🞏 Yes

🞏 No

**Immunomagnetic concentration and separation**

🞏 Yes

🞏 No

**Detection**

*Detection type*

🞏 Presence/absence 🞏 Quantitative

*Molecular methods used:* 🞏 Yes 🞏 No

*If Yes:*

🞏 Extraction method

🞏 qPCR

🞏 Baker assay

🞏 S. Nair quadruplex (PHE)

🞏 Other target gene ______________

🞏 TAC

🞏 Sequencing

🞏 Other ______________________________

Number of replicates per sample

Primers and probe suppliers

Chemistry used (*e.g.,* TaqMan, SYBR green)

Instruments used (*e.g.,* Biorad CFX, Abi7500, NextSeq)

*Culture methods used:* 🞏 Yes 🞏 No

*If Yes:*

Sample pre-enriched prior to:

🞏 Enrichment 🞏 Plating 🞏 PCR

- Sample directly plated

**Cost considerations** *(Costs for single sample)*

Estimated overall ES budget

Estimated cost per ES sample

*(please include estimates for field workers, travel, cold chain [if any], lab processing and storing, and detection)*

🞏 Recurring costs:

🞏 Capital costs:

🞏 Capital equipment purchased for this study?

🞏 Yes *(describe):*

🞏 No

*Personnel*

🞏 Number of field teams:

🞏 Technicians per field team:

🞏 Number of laboratory analysts:

🞏 Number of data analysts:

*Field*

|  | Cost (per sample) | Items included |
| --- | --- | --- |
| Reusable sampling supplies |  |  |
| Disposable sampling supplies |  |  |
| Labor |  |  |
| Transportation |  |  |

*Laboratory*

Labor costs (per sample):

|  | Supplies cost (per sample) | |
| --- | --- | --- |
|  | Reusable | Disposable |
| Concentration |  |  |
| IMS |  |  |
| Enrichment/plating |  |  |
| DNA extraction |  |  |
| Molecular method |  |  |

Items included in costs:

Equipment required for sample processing:

🞏 Centrifuge (*specify vol. capacity)*: mL

🞏 Shaker 🞏 Pump

🞏 Refrigerator 🞏 Biosafety cabinet

🞏 Other *(specify)*:

**Throughput considerations**

*Field*

🞏 Number samples collected in a day:

*Laboratory*

🞏 Number samples processed in a day:

🞏 Total time required for concentration: hr

🞏 Total time personnel actively work with sample (*e.g.,* pH adjustment): hr

🞏 Total time personnel do not actively work with sample (*e.g.,* shaking): hr

*Total time required for (active and inactive time):*

🞏 Enrichment/plating: hr

🞏 DNA extraction: hr

🞏 Sequencing: hr

🞏 Data entry: hr

🞏 Collection to final result: days

🞏 Other: hr

**Sampling Scheme**

Are you conducting clinical surveillance of blood culture samples?

🞏 Yes 🞏 No

*If Yes:*

Are you relating environmental sample results to clinical sample results?

🞏 Yes *(describe approach):*

🞏 No

Is the sampling scheme designed to be representative of the population under surveillance?

🞏 Yes

🞏 No *(describe the sampling scheme in two sentences)*:

*Surveillance population*

🞏 Average catchment population:

🞏 Are there heterogeneities in the population that are accounted for in the sampling scheme? (*e.g.*, differential vaccination rates, differential open-defecation rates, etc.)

🞏 Yes *(describe):*

🞏 No, there are no heterogeneities in the population

🞏 No, heterogeneities in the population are not accounted for
