# Supplementary material for: Estimates of the cost to build a stand-alone environmental surveillance system for typhoid in low- and middle-income countries
Source: PLOS Glob Public Health. 2023 Jan 26;3(1):e0001074. doi: 10.1371/journal.pgph.0001074 (PMC10021573; doi:10.1371/journal.pgph.0001074)
Supplement: S3 Text — Table A in S3 Text. Total and operational costs per sample (modeled). (DOCX) [file pgph.0001074.s003.docx]

# **S3 Text: Total and Operational Costs per Sample**

**Table A.** Total and operational costs per sample at selected levels of scale and for each protocol considered. Annualized costs per sample represent the median, 25^th^, and 75^th^ percentile results from 1,000 simulation runs. All values are in 2019 US dollars.

| Protocol | Site Count, Sampled Bi-Weekly | Annualized cost per sample  USD Median (25^th^, 75^th^) | Operational cost per sample  USD Median (25^th^, 75^th^) |
| --- | --- | --- | --- |
| DC-E-C-qPCR | 25 | 437 (397, 478) | 279 (253, 309) |
| DC-E-C-qPCR | 45 | 264 (240, 290) | 170 (153, 186) |
| DC-E-C-qPCR | 65 | 200 (182, 219) | 129 (117, 143) |
| DC-E-C-qPCR | 125 | 141 (127, 157) | 93 (83, 105) |
| DC-qPCR | 25 | 357 (321, 401) | 218 (192, 249) |
| DC-qPCR | 45 | 216 (195, 241) | 133 (118, 151) |
| DC-qPCR | 65 | 163 (148, 188) | 101 (90, 117) |
| DC-qPCR | 125 | 116 (102, 133) | 74 (64, 85) |
| DEUF-E-qPCR | 25 | 794 (744, 840) | 584 (549, 615) |
| DEUF-E-qPCR | 45 | 665 (626, 705) | 502 (480, 532) |
| DEUF-E-qPCR | 65 | 605 (575, 642) | 467 (447, 490) |
| DEUF-E-qPCR | 125 | 532 (504, 561) | 421 (403, 441) |
| FC-qPCR | 25 | 542 (495, 595) | 366 (334, 407) |
| FC-qPCR | 45 | 411 (365, 468) | 283 (253, 325) |
| FC-qPCR | 65 | 339 (305, 384) | 239 (214, 270) |
| FC-qPCR | 125 | 259 (235, 297) | 188 (169, 214) |
| FC-qPCR_TAC | 25 | 612 (560, 665) | 417 (382, 456) |
| FC-qPCR_TAC | 45 | 474 (429, 524) | 328 (298, 367) |
| FC-qPCR_TAC | 65 | 401 (363, 450) | 281 (257, 319) |
| FC-qPCR_TAC | 125 | 316 (290, 354) | 229 (212, 256) |
| GE-E-qPCR | 25 | 363 (322, 417) | 230 (201, 270) |
| GE-E-qPCR | 45 | 222 (198, 251) | 144 (127, 168) |
| GE-E-qPCR | 65 | 169 (150, 192) | 111 (99, 129) |
| GE-E-qPCR | 125 | 124 (110, 140) | 86 (76, 97) |
| MF-E-C-qPCR | 25 | 501 (454, 554) | 333 (301, 372) |
| MF-E-C-qPCR | 45 | 313 (288, 346) | 215 (196, 238) |
| MF-E-C-qPCR | 65 | 244 (224, 270) | 171 (156, 189) |
| MF-E-C-qPCR | 125 | 179 (163, 197) | 129 (118, 143) |
| MF-E-qPCR | 25 | 490 (444, 541) | 328 (292, 361) |
| MF-E-qPCR | 45 | 308 (282, 338) | 210 (192, 231) |
| MF-E-qPCR | 65 | 239 (220, 262) | 167 (154, 184) |
| MF-E-qPCR | 125 | 178 (162, 196) | 129 (117, 141) |
| MF-qPCR | 25 | 472 (429, 523) | 313 (281, 348) |
| MF-qPCR | 45 | 293 (268, 322) | 199 (181, 218) |
| MF-qPCR | 65 | 225 (207, 249) | 156 (143, 173) |
| MF-qPCR | 125 | 164 (149, 181) | 117 (108, 129) |
| MS-E-qPCR | 25 | 352 (316, 395) | 219 (194, 252) |
| MS-E-qPCR | 45 | 212 (193, 238) | 136 (122, 154) |
| MS-E-qPCR | 65 | 162 (146, 182) | 106 (95, 119) |
| MS-E-qPCR | 125 | 118 (105, 135) | 81 (71, 92) |
| TFUF-E-C-qPCR | 25 | 554 (514, 593) | 382 (353, 410) |
| TFUF-E-C-qPCR | 45 | 383 (357, 408) | 273 (256, 292) |
| TFUF-E-C-qPCR | 65 | 315 (293, 338) | 231 (214, 246) |
| TFUF-E-C-qPCR | 125 | 257 (238, 274) | 194 (180, 208) |
| TFUF-E-qPCR | 25 | 559 (516, 602) | 385 (356, 417) |
| TFUF-E-qPCR | 45 | 381 (355, 406) | 272 (253, 289) |
| TFUF-E-qPCR | 65 | 313 (293, 338) | 229 (214, 247) |
| TFUF-E-qPCR | 125 | 255 (234, 273) | 192 (177, 206) |
| TFUF-qPCR | 25 | 542 (501, 585) | 372 (345, 401) |
| TFUF-qPCR | 45 | 370 (344, 396) | 264 (246, 283) |
| TFUF-qPCR | 65 | 305 (283, 327) | 222 (207, 238) |
| TFUF-qPCR | 125 | 243 (226, 264) | 184 (171, 198) |
