# Supplementary material for: Estimates of the cost to build a stand-alone environmental surveillance system for typhoid in low- and middle-income countries
Source: PLOS Glob Public Health. 2023 Jan 26;3(1):e0001074. doi: 10.1371/journal.pgph.0001074 (PMC10021573; doi:10.1371/journal.pgph.0001074)
Supplement: S4 Text — (DOCX) [file pgph.0001074.s004.docx]

# **S4 Text: Cost Model Details**

The total cost model was built per the following formulaic description.

The cost components are structured as follows, where B = binary (zero or one) depending on whether this cost component exists for a given protocol, m = method, M = the number of methods, uc = unit cost, S = the number of samples, and Q = the number of pieces of equipment. Different equipment, labor roles, and consumables are required for different ES methods and only those appropriate for a protocol are costed.

$$Sample Days=SD=Number of samples that are collected from a given site, annually=26$$

$$S_{annual}=Number of Sites*SD$$

$$S_{ptpd,m}=Average daily samples collected (per team per day), for a given method$$

$$Sites per Team per Year=\frac{S_{ptpd,m}*5 days per week*46 workweeks per year}{SD}$$

$$Number of Field Teams=NFT= ceiling (\frac{Number of sites}{Sites per Team per Year} )$$

$$Truck Purchase= {uc}_{truck}*NFT$$

$$Truck Depreciation= \frac{Truck Purchase}{lifespan}$$

$$Truck Operations= Truck Depreciation*operations rate$$

$$Truck Maintenance= Truck Depreciation*maintenance rate$$

$$Equipment Purchase= \sum_{i=1}^{Q} {Equipment}_{i}=\sum_{i=1}^{Q} {uc}_{i}*B_{m}*ceiling\left( \frac{S_{annual}}{{capacity}_{i}} \right)$$

$$Equipment Maintenance=\sum_{i=1}^{Q} {Equipment Purchase}_{i}* {rate}_{maintenance,i}$$

$$Equipment Depreciation= \sum_{i=1}^{Q} \frac{{Equipment Purchase}_{i}}{{lifespan}_{i}}$$

$$Disposables= {uc}_{m} *B_{m}*S_{annual}$$

$Reusables= {uc}_{m,annual\_per\_team}*B_{m}*NFT+ {uc}_{m,daily\_per\_team}*B_{m}*{ceiling(\frac{S_{annual}}{S}}_{ptpd})$

$$Consumables= Reusables+Disposables$$

$$Collection Labor= NFT*Techs per Team*{uc}_{tech\_dayrate}* SD$$

$$Laboratory Time per Day= LTPD=\sum_{m=1}^{M} B_{m}*ceiling\left( \frac{S_{daily}}{{Batch Size}_{m}} \right)* {labor time per batch}_{m}$$

$$Laboratory Labor= {uc}_{lab staff}*ceiling(\frac{LTPD}{(46 weeks per year*40 hours per week*.80 availability)})$$

$$Labor=Collection Labor+Laboratory Labor$$

$$Operational= Labor+Consumables+Equipment Maintenance+Truck Maintenance+ Truck Operations$$

$$Overhead= {rate}_{management}*Labor+ {rate}_{building}*Operational$$

$$Total=Equipment Purchase+Operational+Overhead$$
