# Supplementary material for: Estimates of the cost to build a stand-alone environmental surveillance system for typhoid in low- and middle-income countries
Source: PLOS Glob Public Health. 2023 Jan 26;3(1):e0001074. doi: 10.1371/journal.pgph.0001074 (PMC10021573; doi:10.1371/journal.pgph.0001074)
Supplement: Attachment — Submitted filename: Response to Reviewers.docx [file pgph.0001074.s005.docx]

Journal Requirements:

1. We have amended your Competing Interest statement to comply with journal style. We kindly ask that you double check the statement and let us know if anything is incorrect. We have verified that this is correct.

2. Please amend your detailed Financial Disclosure statement. This is published with the article. It must therefore be completed in full sentences and contain the exact wording you wish to be published. We have edited the financial disclosure statement to more clearly detail the salaries received by BH and JG, and updated the text to initials per the journal’s formatting requirements. We have also added the statement that the funders had no role in the manuscript.

3. Please provide the full and correct funding information for your study and confirm the order in which funding contributions should appear. We have updated the funding statement per the explanation on point #2 above and confirm that this is correctly stated.

4. We have noticed that you have uploaded Supporting Information files, but you have not included a list of legends. Please add a full list of legends for your Supporting Information files after the references list. We have added a detailed list of supplement content to the manuscript, after the references.

5. In the online submission form you indicate that your data is not available for proprietary reasons and have provided a contact point for accessing this data. Please note that your current contact point is a co-author on this manuscript. According to our Data Policy, the contact point must not be an author on the manuscript and must be a third party. Please revise your data statement to a non-author institutional point of contact, such as a data access or ethics committee, and send this to us via return email. Please also include contact information for the third party organization, and please include the full citation of where the data can be found. We have updated the data availability statement to clarify the reason that the individual data points are not available. We have also added the sample size (n) to the supplement Table S1.3 to make the small sample size more apparent for certain data points. We also added details on how the cost survey data were used to generate the distributions in the description; this information is also in the manuscript, but we felt that for clarity it was helpful to include in the supplement as well.

Please review your reference list to ensure that it is complete and correct. If you have cited papers that have been retracted, please include the rationale for doing so in the manuscript text, or remove these references and replace them with relevant current references. Any changes to the reference list should be mentioned in the rebuttal letter that accompanies your revised manuscript. If you need to cite a retracted article, indicate the article’s retracted status in the References list and also include a citation and full reference for the retraction notice. We have completed a review and no changes were required.

Editorial comments:

Based on the analysis and results, given there is heterogeneity in the annualized cost across surveillance systems, especially with reference to labour, equipment and reagents - it would be helpful to provide clear guidance to the readers on approaches to setting up new surveillance systems under certain constraints such as high labour cost versus high equipment cost versus high recurring costs in LMICs settings.

We agree with the editor that this was not as clear as it could have been in the manuscript. We have added a paragraph to the discussion describing how a reader could use the information found in Figure 3 on the relative contribution of cost categories, to assess the best choice for them in the context of their local economy.

Reviewer #1:

The manuscript gives detailed estimation of cost for thirteen protocols used in six countries. It is an important and timely study particularly when most of the countries are exploring to invest in newer surveillance methods like environmental surveillance systems.

Thank you, we agree this can be valuable for evaluating investments in surveillance systems.

However, it will be more useful for policymakers in the LMICs if the authors can give recommendation on cost-effective protocol with respect to the sensitivity of the protocol. Future studies can also be recommended if needed.

This study does not assess cost-effectiveness because the use cases for how the results will be integrated into policy decision making is not yet clear, which makes any cost-effectiveness analysis quite theoretical at this stage in the development of this technology. For example, the relative value of a quantified level versus presence/absence of a pathogen is not yet established and could make some protocols more beneficial to policy makers and thus change the cost-effectiveness calculations.

We hope to see future work on this question, which can be built upon the cost estimates we provide here. We have added to the discussion section to make this point and recommend future studies that will be able to provide clarity on this question.

It will add more value if authors can clarify on how the labor market dynamics from the LMICs are taken care of in calculating the average cost.

Thank you for this question. The reviewer is correct that the local labor markets can vary widely and we see this in the reported day labor rates from the costing surveys. These details are reported in Table S2.1 and we have emphasized this as a key source of uncertainty by adding a paragraph in the methods section about the market variability and how we derived the values used in these estimates.
